# Longer Diagnostic Delay and Post-Diagnosis Overall Survival in a Classic-Kaposi-Sarcoma-Predominant Retrospective Cohort

**DOI:** 10.3390/jcm15114243

**Published:** 2026-05-30

**Authors:** İsmail Bayrakçı, Didem Divriklioğlu, Gizem Bakır Kahveci, Tayyip İlker Aydın, Dicle Yurdatap Koç, Ece Demirdelen, Ahmet Küçükarda, Muhammet Bekir Hacıoğlu, Bülent Erdoğan, Sernaz Topaloğlu

**Affiliations:** Department of Internal Medicine, Division of Medical Oncology, Trakya University Faculty of Medicine, Edirne 22030, Türkiye; dr_didemeroglu@hotmail.com (D.D.); bakirkahvecigizem@gmail.com (G.B.K.); ilker6125@gmail.com (T.İ.A.); dicleyurdatap@gmail.com (D.Y.K.); demirdelen_ece@hotmail.com (E.D.); ahmetkucukarda22@gmail.com (A.K.); mbekirhacioglu@yahoo.com (M.B.H.); berdoga@hotmail.com (B.E.); sernaz.uzunoglu@gmail.com (S.T.)

**Keywords:** Kaposi sarcoma, diagnostic delay, overall survival, progression-free survival, maxstat, retrospective cohort

## Abstract

**Background/Objectives:** Kaposi sarcoma (KS) is an HHV-8-associated angioproliferative malignancy with heterogeneous clinical presentation. Early lesions may be overlooked or misattributed to benign conditions, potentially causing diagnostic delay. We evaluated whether diagnostic delay was associated with survival outcomes in a real-world KS cohort. **Methods:** We retrospectively analyzed 87 consecutive patients with histologically confirmed KS diagnosed between 2007 and 2025. Diagnostic delay was defined as the interval between first patient-reported lesion/symptom recognition documented in medical records and histological diagnosis. An exploratory, maximally selected log-rank method was used to identify the delay cut-off that best separated overall survival (OS). OS and progression-free survival (PFS) were analyzed using Kaplan–Meier estimates, log-rank tests, and Cox regression models. **Results:** The exploratory optimal cut-off for diagnostic delay was 6.5 months, defining early (≤6.5 months; *n* = 46) and late (>6.5 months; *n* = 41) diagnosis groups. Median OS was 202.2 months (95% CI 62.3–342.1) in the early group and 92.9 months (95% CI 61.8–124.0) in the late group (log-rank *p* < 0.001). Late diagnosis was associated with a higher risk of death (HR 3.6, 95% CI 1.6–8.1; *p* = 0.001). This association was attenuated after multivariable adjustment and was no longer statistically significant (adjusted HR, 1.711, 95% CI 0.696–4.207; *p* = 0.242). Patients with late diagnosis were older (median 74 vs. 67 years, *p* = 0.004), had greater comorbidity burden (39.0% vs. 13.0%; *p* = 0.005), and more frequently had lymphedema (19.5% vs. 4.3%; *p* = 0.041). **Conclusions:** In this single-center KS cohort, longer diagnostic delay was associated with poorer post-diagnosis overall survival in unadjusted analyses, while this association was attenuated after multivariable adjustment. The exploratory 6.5-month threshold identified a subgroup with less favorable survival; however, this data-driven cut-off should be considered hypothesis-generating. These findings support efforts to improve early recognition, biopsy, and referral in KS, particularly in older and more comorbid patients.

## 1. Introduction

Kaposi sarcoma (KS) is a multicentric, angioproliferative neoplasm driven by human herpesvirus-8 (HHV-8/KSHV), with four major clinical–epidemiologic subtypes—classic, endemic/African, iatrogenic, and epidemic/HIV-related [1,2]. Clinically, KS most commonly presents as violaceous macules, plaques, or nodules, frequently involving the distal extremities; symptoms such as edema/lymphedema, pain, bleeding, and ulceration may accompany cutaneous disease and are more likely to occur with increasing disease burden [1,3,4]. Importantly, KS can involve mucosal surfaces, lymph nodes, and visceral organs, and the outcome is linked to the extent of disease and systemic involvement [1,5]. Across real-world cohorts, classic KS typically affects older men, often with lower-extremity predominance, while HIV-associated cases present at younger ages and may show higher extracutaneous involvement, highlighting how immune context and epidemiology shape presentation and prognosis [1,5].

Prognostication in KS has traditionally emphasized tumor burden and distribution, particularly in HIV-associated KS. Risk stratification frameworks (e.g., ACTG/TIS) are clinically meaningful because advanced disease often requires chemotherapy in addition to antiretroviral therapy [6]. In non-HIV-related cohorts, progression and survival signals have been linked to patient factors (age, immunosuppression/comorbidity) and disease extent; large classic KS series have shown that older age and immunosuppression are associated with poorer outcomes [7]. In Mediterranean/North African cohorts, advanced stages, lymphedema, ulceration, and visceral spread are repeatedly encountered, underscoring that classic KS is not always purely indolent in real life [3]. Together, these observations suggest that more advanced presentation tends to coexist with less favorable outcomes across settings, even though the phenotype, care pathways, and treatment resources differ by region and subtype [3,6,7].

Diagnostic delay is one potentially modifiable contributor to advanced presentation and may arise both before first healthcare contact and within the health system (referral, biopsy availability, and pathology turnaround). In Uganda, among HIV-infected adults with histologically confirmed KS, diagnostic delay was common and was associated with poor-risk stage at diagnosis [8]. In western Kenya, qualitative interviews identified both patient-level barriers (low KS awareness, fear of cancer/biopsy/amputation, stigma, reliance on traditional treatments) and health-system barriers (incorrect diagnoses, incomplete physical examination, delayed referral, lack of biopsy services) that may prolong time to diagnosis [6]. Even in classic KS settings, substantial time to consultation can exist; for example, a Moroccan classic KS series reported a median 12-month duration from first lesion appearance to consultation [3]. Taken together, these studies support the plausibility that delayed diagnosis may contribute to more advanced presentation and potentially poorer outcomes, while also showing that delay may cluster with broader patient- and system-level vulnerabilities.

Despite this rationale, the survival implications of diagnostic delay remain insufficiently characterized in real-world KS cohorts, particularly those not limited to HIV-associated disease. Therefore, in this single-center retrospective cohort, we evaluated diagnostic delay in patients with KS and examined its association with survival outcomes. We also explored whether delayed diagnosis was associated with baseline clinical features that may reflect vulnerability to delayed evaluation.

## 2. Materials and Methods

### 2.1. Study Design and Setting

This was a single-center retrospective cohort study including patients diagnosed with KS between 2007 and 2025 at the Department of Medical Oncology, Trakya University Faculty of Medicine. The study aimed to (i) describe demographic, clinical, and treatment characteristics and (ii) evaluate whether diagnostic delay is associated with survival outcomes and baseline disease phenotype. The study was reported following STROBE recommendations for observational studies [9].

### 2.2. Participants and Eligibility

We included all consecutive patients with histopathologically confirmed KS who were treated and/or followed at our center during the study period (*n* = 87). Patients were categorized by epidemiologic subtype as classic KS (*n* = 78), iatrogenic/transplant- or immunosuppression-related KS (*n* = 3), and HIV-associated KS (*n* = 6). Because of the limited number of non-classic cases, a unified extent-based staging approach, as recorded in the institutional database, was used across subtypes for descriptive and comparative analyses. For consistency, disease extent was reported in three categories throughout the manuscript: stage I–II (localized disease), stage III (locally advanced disease), and stage IV (metastatic/visceral disease). This pragmatic approach was chosen to allow analysis of the full cohort, although biologic and prognostic differences between KS subtypes were acknowledged.

### 2.3. Data Sources and Variables

Data were extracted from patient files and the hospital electronic information system. Baseline variables included age, sex, date of diagnosis, lesion localization, stage at diagnosis, clinical morphology, second malignancy, HHV-8/LANA status (if available), comorbidities, and smoking status. Treatment modalities were recorded in detail. Follow-up data included relapse/progression events and the last follow-up date. Comorbidity burden was summarized using the Charlson Comorbidity Index.

### 2.4. Exposure Definition: Diagnostic Delay

Diagnostic delay was defined as the time interval between the first patient-reported recognition of a lesion or symptom onset, as documented in the clinical history, and the date of histological diagnosis. This variable was derived retrospectively from routine clinical documentation rather than from a prospectively standardized symptom diary or questionnaire. Extracted diagnostic-delay data were checked for chronological consistency during data cleaning by the study investigators. However, formal independent duplicate abstraction or blinded validation was not performed because of the retrospective design. Because approximate onset descriptions were not prospectively coded as a separate variable, a sensitivity analysis excluding these cases could not be reliably performed. Accordingly, diagnostic delay was considered a clinically relevant but potentially imprecise exposure, reflecting both patient recall and the quality of documentation. Because onset timing was based on retrospective clinical records and patient-reported history, the possibility of recall bias and non-standardized measurement was acknowledged in the interpretation of the findings. When the timing of symptom or lesion onset was not precisely documented, the variable was derived from the available clinical record.

### 2.5. Outcomes

The primary outcome was overall survival (OS), defined as the time from histological diagnosis to death from any cause or last follow-up. The secondary outcome was progression-free survival (PFS), defined as the time from diagnosis to documented progression or relapse, death, or last follow-up without an event. Other response endpoints included best overall response, objective response rate, and disease control rate. In this retrospective cohort, response and progression assessments were based on treating-physician documentation, available clinical examination findings, imaging results when performed, and pathology reports when applicable, rather than on a prespecified prospective protocol such as RECIST.

### 2.6. Statistical Analysis

All analyses were performed using R version 4.5.2 and SPSS version 25 (IBM Corp., Armonk, NY, USA). Continuous variables are reported as mean (standard deviation) or median (Q1–Q3), as appropriate. Categorical variables are presented as a number (%). Group comparisons were performed using appropriate parametric or nonparametric tests based on variable type and distribution.

Diagnostic delay was examined as a time-based exposure variable. Because no validated clinical threshold for diagnostic delay was available in this cohort, an exploratory maximally selected log-rank procedure implemented in the R ‘maxstat’ package was used to identify the delay cut-off that best separated overall survival [10,11]. Diagnostic delay was evaluated as the candidate continuous variable, and overall survival was used as the survival endpoint. The procedure identifies the cut-point that maximizes the separation of survival curves across candidate thresholds. The resulting 6.5-month cut-off was considered data-driven, exploratory, and hypothesis-generating rather than a validated clinical threshold. Accordingly, post-selection log-rank *p*-values were interpreted descriptively and not as confirmatory evidence. Patients were then categorized as having early (≤6.5 months) or late (>6.5 months) diagnosis for descriptive, comparative, and survival analyses.

During routine clinical evaluation, symptom or lesion onset was frequently documented using approximate patient-reported durations rather than exact calendar dates. In such cases, onset timing was retrospectively estimated from the reported duration relative to the clinical presentation date. Therefore, diagnostic delay was considered a clinically meaningful but potentially approximate exposure variable.

OS and PFS were estimated using the Kaplan–Meier method and compared with the log-rank test. Prognostic factors for OS and PFS were evaluated using univariate and multivariable Cox proportional hazards regression analyses in R (survival package). Penalized Cox regression with Firth correction was applied when standard Cox models yielded unstable or non-estimable estimates because of sparse data or separation [12,13]. The proportional hazards assumption was assessed using Schoenfeld residuals, and no significant violations were detected. Candidate variables for multivariable modeling included demographic, laboratory, clinical, treatment-related, and comorbidity-related parameters. Multivariable models were constructed using a parsimonious approach that considered clinical relevance, prior literature, model interpretability, and univariate findings, while limiting model complexity because of the sample size and number of events. Diagnostic delay, the main exposure of interest, was then included in the final parsimonious models to assess its associations with OS and PFS. As a sensitivity analysis, diagnostic delay was additionally evaluated as a continuous variable in a univariate Cox regression model. Adjusted OS and PFS probabilities were derived from the final multivariable Cox proportional hazards models using the SPSS survival table output based on survival estimates at the mean of covariates. No missing data were present for the variables included in the final regression analyses. However, some treatment-related variables were available only within relevant clinical subgroups because of the retrospective cohort design.

### 2.7. Ethics Statement

This study was conducted in accordance with the Declaration of Helsinki. Ethical approval was obtained from the Trakya University Faculty of Medicine Clinical Intervention Ethics Committee (approval no: TÜ TF-GOBAEK 2025/470, date: 3 November 2025). Because of the retrospective design and the use of de-identified data, the requirement for informed consent was waived.

## 3. Results

### 3.1. Patient Characteristics

A total of 87 consecutive patients with histopathologically confirmed KS were included in the study. Demographic, clinical, and treatment characteristics according to diagnostic delay group are summarized in Table 1. The cohort was predominantly composed of patients with classic KS, and lower-extremity involvement was the most common presentation.

### 3.2. Diagnostic Delay Cut-Off and Overall Survival

**Figure 1:**
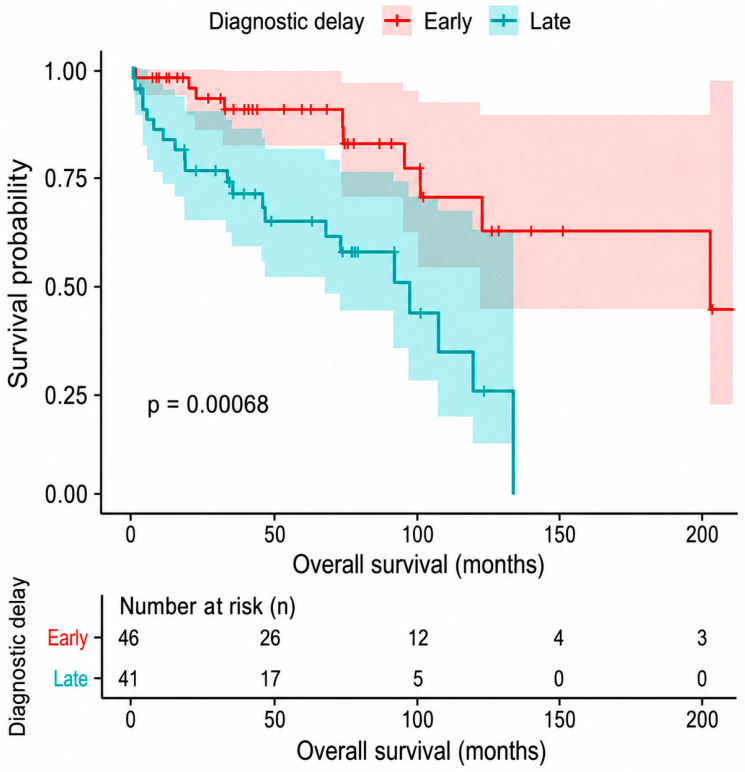
Kaplan–Meier overall survival curves according to diagnostic-delay group. Patients were categorized as having early diagnosis (≤6.5 months) or late diagnosis (>6.5 months) according to the exploratory maxstat-derived diagnostic-delay cut-off. The shaded areas represent 95% confidence intervals. The number-at-risk table is shown below the survival curves. The *p*-value represents the log-rank comparison between groups and should be interpreted descriptively in the context of the exploratory data-driven cut-off.

Using an exploratory maximally selected log-rank procedure, the optimal diagnostic delay cut-off for separating overall survival (OS) was identified as 6.5 months, and is shown in Table 2. Based on this threshold, patients were categorized as having an early diagnosis (≤6.5 months; *n* = 46) or a late diagnosis (>6.5 months; *n* = 41) (Table 2). Median OS was 202.2 months (95% CI 62.3–342.1) in the early diagnosis group and 92.9 months (95% CI 61.8–124.0) in the late diagnosis group. This difference was statistically significant in Kaplan–Meier analysis (log-rank *p* < 0.001). In the corresponding Cox model, late diagnosis was associated with a higher risk of death (HR 3.6, 95% CI 1.6–8.1; *p* = 0.001). In this cohort, 31 death events were observed during follow-up. The Kaplan–Meier overall survival curves by diagnostic-delay group are shown in Figure 1, along with the number-at-risk table.

One-, three-, five-, and ten-year OS estimates are provided in Supplementary Table S1.

### 3.3. Comparison of Early Versus Late Diagnosis Groups

Patients in the late diagnosis group were significantly older than those in the early diagnosis group and had a greater comorbidity burden. Lymphedema was also more frequent among patients with late diagnosis. Most other baseline clinical features, including overall disease stage, were broadly similar between groups (Table 1). Treatment response rates were also broadly comparable. Progression-free survival was assessed using Kaplan–Meier analysis and Cox regression, rather than descriptive group comparisons. Diagnostic delay was not significantly associated with PFS in these time-to-event analyses. Table 1 also presents treatment-related and response variables according to the diagnostic delay group.

### 3.4. Univariate Cox Regression Analyses

In univariate Cox regression analyses, late diagnosis (>6.5 months vs. ≤6.5 months) was significantly associated with worse OS (HR 3.637, 95% CI 1.644–8.104; *p* = 0.001). When evaluated as a continuous variable, diagnostic delay was not significantly associated with overall survival (HR 1.020 per month increase, 95% CI 0.994–1.047; *p* = 0.136).

Increasing age was also associated with inferior OS, both when modeled continuously (HR 1.115, 95% CI 1.068–1.165; *p* < 0.001) and when dichotomized at 70 years (HR 4.524, 95% CI 2.042–10.024; *p* < 0.001). Higher comorbidity burden was likewise associated with poorer OS in several pairwise comparisons.

A total of 43 progression/relapse events were observed, while 44 patients had no documented PFS event during follow-up. For PFS, male sex (HR 2.421, 95% CI 1.108–5.293; *p* = 0.027) and increasing age (HR 1.042, 95% CI 1.010–1.075; *p* = 0.011) were associated with worse outcomes, whereas late diagnosis was not significantly associated with PFS (HR 1.543, 95% CI 0.837–2.841; *p* = 0.165) (Table 3).

### 3.5. Multivariable Cox Regression Models

Multivariable Cox regression models were fitted separately for OS and PFS (Table 4). In the adjusted OS model, male sex, age > 70 years, lower hematocrit, and visceral organ involvement remained independently associated with worse OS. Diagnostic delay, when entered into the adjusted OS model based on the exploratory 6.5-month threshold, was attenuated and was no longer statistically significant. In the adjusted PFS model, male sex, multifocal cutaneous distribution, mucosal involvement, and more advanced overall disease stage remained independently associated with poorer PFS, whereas diagnostic delay was not significantly associated with PFS. Adjusted OS and PFS probabilities at 1, 3, 5, and 10 years are shown in Table 4.

## 4. Discussion

Our principal finding is that diagnostic delays of more than approximately 6.5 months were associated with poorer overall survival in unadjusted analyses in this real-world, single-center KS cohort. Using an exploratory data-driven approach, a 6.5-month threshold identified a subgroup with shorter post-diagnosis OS. However, this association was attenuated after adjustment for key covariates, suggesting that diagnostic delay may reflect a broader pattern of clinical vulnerability rather than acting as an independently confirmed prognostic factor in this dataset. Importantly, patients in the late-diagnosis group were older and had a higher comorbidity burden, indicating that delay may cluster among clinically vulnerable patients and may interact with frailty-related barriers to timely assessment and biopsy [6,7,8].

Our results are broadly consistent with previous reports linking diagnostic delay to less favorable presentation patterns, even when the reported endpoints differ across studies. In Uganda, diagnostic delay was associated with a poor-risk stage at diagnosis, and potentially modifiable contributors included care-seeking behaviors, such as consultations with traditional healers [8]. In western Kenya, qualitative interviews mapped delays across the diagnostic pathway, identifying both patient-level barriers (e.g., limited awareness, fear, stigma, and reliance on traditional treatment) and health-system barriers (e.g., misdiagnosis, incomplete examination, delayed referral, and limited biopsy availability), further supporting the plausibility that delay may translate into stage shift and poorer outcomes [6]. Even in classic KS settings, prolonged time to consultation has been documented; for example, a Moroccan cohort reported a median 12-month interval between first lesion appearance and consultation, underscoring that diagnostic delay is not unique to HIV-endemic contexts [3]. Taken together, these observations support the plausibility that prolonged diagnostic delay may contribute to more advanced presentation and potentially less favorable outcomes across different KS settings, while also reflecting broader patient and system vulnerabilities.

Interestingly, diagnostic delay showed a strong association with OS but not with PFS in univariate analysis, whereas adjusted PFS models were driven primarily by disease extent and biology, including mucosal involvement, multifocal distribution, and stage. One possible explanation is that the OS in this cohort may be more sensitive to overall vulnerability and competing risks, as late-diagnosed patients were older and had a greater comorbidity burden. By contrast, PFS may directly reflect tumor behavior once patients enter structured follow-up and treatment pathways. In addition, in classic KS, progression events may be influenced by surveillance intensity and treatment patterns, potentially making PFS less sensitive to diagnostic timing alone. These interpretations should be viewed as hypothesis-generating rather than definitive, given the retrospective design and limited sample size. These considerations may help explain why diagnostic delay was associated with overall survival but not progression-free survival in this cohort.

From a clinical and public health perspective, our findings suggest several actionable opportunities to reduce diagnostic delay, including greater provider awareness of KS among vascular-appearing lesions, a low threshold for biopsy with rapid communication of pathology results, and streamlined referral pathways, particularly for older and comorbid patients who may face access barriers. These implications mirror intervention targets found in pathway-based qualitative work, in which improving awareness, biopsy availability, and referral efficiency were highlighted as key levers [6]. At the same time, our study has limitations typical of retrospective cohorts. The diagnostic delay variable was based on patient-reported timing documented in medical records and is therefore subject to recall and documentation biases. Any resulting misclassification would be expected to be largely non-differential and therefore more likely to attenuate than systematically exaggerate the observed associations. In addition, because diagnostic delay was defined before histological diagnosis, whereas OS and PFS were measured from the date of diagnosis, lead-time bias should be considered when interpreting the survival findings. A formal reverse Kaplan–Meier median follow-up estimate could not be reliably calculated because of the retrospective nature of the dataset and limitations of follow-up documentation. Therefore, long-term survival estimates should be interpreted cautiously, particularly at later time points when the number of patients at risk declined substantially. The 6.5-month cut-off was identified using a data-driven exploratory approach and may be vulnerable to overfitting; therefore, it should not be interpreted as a definitive clinical threshold and requires validation in independent cohorts. Moreover, diagnostic delay was not significantly associated with OS when modeled as a continuous variable, further supporting the interpretation that the 6.5-month cut-off should be viewed as exploratory rather than definitive. Finally, as a single-center study with a predominantly classic KS population and only a small number of HIV-related and iatrogenic cases, the generalizability of our findings to other practice settings and KS subtypes is limited. Accordingly, the results should be interpreted primarily in the context of a classic-KS-predominant cohort, rather than generalized equally across all epidemiologic forms of KS. Future multicenter studies should validate this threshold and further disentangle patient-related and health-system-related components of diagnostic delay.

## 5. Conclusions

In this single-center real-world cohort of KS, longer diagnostic delay was associated with poorer post-diagnosis overall survival in unadjusted analyses. An exploratory 6.5-month threshold identified a subgroup with less favorable survival, although this data-driven cut-off requires external validation. Because the observed association was attenuated after multivariable adjustment and may be influenced by lead-time bias and residual confounding, these findings should be interpreted cautiously. Nevertheless, they support efforts to improve timely recognition, biopsy, and referral pathways for KS, especially among older and more comorbid patients who may be more vulnerable to delayed diagnosis.

## Figures and Tables

**Table 1:** Demographic, clinical, and treatment characteristics according to diagnostic delay group.

		Delay from First Symptom to Histological Diagnosis (m) (Maxstat Cut-Off (6.5 m))			*p*
		Total (*n* = 87)	Early (*n* = 46)	Late (*n* = 41)	
Age, med (Q1–Q3)		69 (64–77)	67 (60–71)	74 (66–79)	**0.004 ^u^**
Sex (female), *n* (%)		22 (25.3)	12 (26.1)	10 (24.4)	0.999 ^c^
Age, *n* (%)					**0.001 ^c^**
	≤70 yr	47 (54.0)	**33 (71.7)**	14 (34.1)	
	>70 yr	40 (46.0)	13 (28.3)	**27 (65.9)**	
Smoking, *n* (%)					0.999 ^c^
	No smoking history	54 (62.1)	29 (63.0)	25 (61.0)	
	Current or former smoking	33 (37.9)	17 (37.0)	16 (39.0)	
Comorbidity Count, *n* (%)					**0.014 ^c^**
	None	16 (18.4)	11 (23.9)	5 (12.2)	
	I	26 (29.9)	18 (39.1)	8 (19.5)	**0.046**
	II	23 (26.4)	11 (23.9)	12 (29.3)	
	≥III	22 (25.3)	6 (13.0)	16 (39.0)	**0.005**
Neutrophil count (×10^3^), mean (SD)		4.7 (1.7)	4.3 (1.6)	5.2 (1.7)	**0.029 ^t^**
Hematocrit, med (Q1–Q3)		38.4 (35.2–42.3)	40.55 (36.1–42.8)	37.8 (34.3–41)	0.116 ^u^
Kaposi Sarcoma subtype, *n* (%)					0.757 ^f^^f^
	Classic	78 (89.7)	40 (87.0)	38 (92.7)	
	Iatrogenic	3 (3.4)	2 (4.3)	1 (2.4)	
	HIV-related	6 (6.9)	4 (8.7)	2 (4.9)	
Disease localization, *n* (%)					0.180 ^f^
	Extracutaneous involvement present	10 (11.5)	3 (6.5)	7 (17.1)	
	Cutaneous only	77 (88.5)	43 (93.5)	34 (82.9)	
Cutaneous presentation, *n* (%)					0.087 ^c^
	Disseminated	36 (41.4)	15 (32.6)	21 (51.2)	
	Localized	51 (58.6)	31 (67.4)	20 (48.8)	
Cutaneous distribution of the lesion, *n* (%)					0.153 ^c^
	Localized	35 (40.2)	23 (50.0)	12 (29.3)	
	Multifocal within the same limb	19 (21.8)	9 (19.6)	10 (24.4)	
	Multicentric	33 (37.9)	14 (30.4)	19 (46.3)	
Clinical lesion morphology, *n* (%)					0.999 ^c^
	Macule/Plaque	47 (54.0)	25 (54.3)	22 (53.7)	
	Nodule/Tumor	40 (46.0)	21 (45.7)	19 (46.3)	
Lymphedema, *n* (%)		10 (11.5)	2 (4.3)	**8 (19.5)**	**0.041 ^f^**
Ulceration, *n* (%)		6 (6.9)	1 (2.2)	5 (12.2)	0.096 ^f^
Visceral organ involvement, *n* (%)		4 (4.6)	1 (2.2)	3 (7.3)	0.339 ^f^
Mucosal involvement, *n* (%)		8 (9.2)	3 (6.5)	5 (12.2)	0.467 ^f^
Overall disease stage, *n* (%)					0.393 ^f^^f^
	Stage I–II	51 (58.6)	30 (65.2)	21 (51.2)	
	Stage III	27 (31.0)	12 (26.1)	15 (36.6)	
	Stage IV	9 (10.3)	4 (8.7)	5 (12.2)	
Primary treatment strategy, *n* (%)					0.790 ^f^^f^
	Local therapy only	62 (71.3)	32 (69.6)	30 (73.2)	
	Systemic therapy only	14 (16.1)	7 (15.2)	7 (17.1)	
	Combined local and systemic therapy	11 (12.6)	7 (15.2)	4 (9.8)	
Type of local treatment, *n* (%)					0.159 ^f^^f^
	Surgery	21 (28.8)	14 (35.9)	7 (20.6)	
	Radiotherapy	39 (53.4)	16 (41.0)	23 (67.6)	
	RT+Excision	9 (12.3)	6 (15.4)	3 (8.8)	
	Cryotherapy and laser therapy	4 (5.5)	3 (7.7)	1 (2.9)	
First-line systemic treatment regimen, *n* (%)					0.730 ^f^^f^
	Liposomal doxorubicin	5 (20.0)	3 (21.4)	2 (18.2)	
	Paclitaxel	17 (68.0)	10 (71.4)	7 (63.6)	
	Interferon-alpha	1 (4.0)	0 (0.0)	1 (9.1)	
	Vinorelbine	1 (4.0)	0 (0.0)	1 (9.1)	
	Etoposide	1 (4.0)	1 (7.1)	0 (0.0)	
Best overall response, *n* (%)					0.291 ^f^^f^
	Complete response (CR)	15 (17.2)	9 (19.6)	6 (14.6)	
	Partial response (PR)	46 (52.9)	24 (52.2)	22 (53.7)	
	Stable disease (SD)	20 (23.0)	12 (26.1)	8 (19.5)	
	Progressive disease (PD)	6 (6.9)	1 (2.2)	5 (12.2)	
Objective Response Rate (ORR), *n* (%)		61 (70.1)	33 (71.7)	28 (68.3)	0.816 ^c^
Disease Control Rate (DCR), *n* (%)		81 (93.1)	45 (97.8)	36 (87.8)	0.096 ^f^
Progressive Disease, *n* (%)		6 (6.9)	1 (2.2)	5 (12.2)	0.096 ^f^
Subsequent-line systemic therapy after progression, *n* (%)					**0.004 ^f^^f^**
	None	13 (30.2)	2 (9.5)	11 (50.0)	**0.004**
	Liposomal doxorubicin	6 (14.0)	5 (23.8)	1 (4.5)	
	Paclitaxel	16 (37.2)	8 (38.1)	8 (36.4)	
	Oral etoposide	1 (2.3)	0 (0.0)	1 (4.5)	
	Radiotherapy	7 (16.3)	6 (28.6)	1 (4.5)	**0.033**

Abbreviations: Diagnostic delay groups were defined using the maxstat-derived 6.5-month cut-off: early diagnosis (≤6.5 months) and late diagnosis (>6.5 months). SD: standard deviation; med: median; m: months. ^u^ Mann–Whitney U Test (Monte Carlo). ^t^ Independent Samples T Test (Bootstrap). ^c^ Pearson Chi-Square Test (Monte Carlo). ^f^ Fisher Exact Test (Monte Carlo). ^f^^f^ Fisher–Freeman–Halton Test (Monte Carlo). Bold signifies the statistically significant *p*-values.

**Table 2:** Exploratory maxstat-derived diagnostic delay cut-off and its association with overall survival.

Diagnostic Delay Cut-Off and Group Distribution		Kaplan–Meier		Cox Regression Analyses	
Cut-Off (m) = 6.5	Exploratory Maxstat *p*-Value	Median OS (m) 95% CI	*p* Value	HR (Late vs. Early)	*p* Value
Early (*n* = 46)	0.016	202.2 (62.3–342.1)	**<0.001**	3.6 (1.6–8.1)	**0.001**
Late (*n* = 41)		92.9 (61.8–124.0)			

Abbreviations: OS: overall survival; HR: hazard ratio; CI: confidence interval; maxstat: maximally selected log-rank statistics; m: months. Diagnostic delay was defined as the interval between first symptom/lesion recognition and histological diagnosis. The 6.5-month cut-off was identified using an exploratory maximally selected log-rank procedure. HR represents the risk of death for late diagnosis (>6.5 months) versus early diagnosis (≤6.5 months). The reported *p*-value corresponds to the exploratory maximally selected log-rank procedure and should be interpreted descriptively rather than as confirmatory statistical evidence. Bold signifies the statistically significant *p*-values.

**Table 3:** Univariate Cox proportional hazards analyses for OS and PFS.

Variable and Comparison		Overall Survival (OS)		Progression-Free Survival (PFS)	
		HR (95% CI)	*p* Value	HR (95% CI)	*p* Value
Sex (Male vs. Female ^r^)		1.443 (0.647–3.216)	0.370	2.421 (1.108–5.293)	**0.027**
Age (Per 1 unit increase)		1.115 (1.068–1.165)	**<0.001**	1.042 (1.010–1.075)	**0.011**
Age (>70 yr vs. ≤70 yr ^r^)		4.524 (2.042–10.024)	**<0.001**	1.782 (0.963–3.297)	0.066
Smoking (No smoking history vs. Current or former smoking ^r^)		0.791 (0.362–1.728)	0.557	0.976 (0.520–1.835)	0.941
Late diagnosis (>6.5 months) vs. early diagnosis (≤6.5 months)		3.637 (1.644–8.104)	**0.001**	1.543 (0.837–2.841)	0.165
Comorbidity Count					
	None vs. I ^r^	1.237 (0.295–5.194)	0.771	1.159 (0.448–3.000)	0.761
	None vs. II ^r^	3.011 (0.827–10.966)	0.095	1.325 (0.513–3.425)	0.561
	None vs. ≥III ^r^	5.635 (1.551–20.463)	**0.009**	2.109 (0.843–5.276)	0.111
	I vs. II ^r^	2.434 (0.825–7.175)	0.107	1.143 (0.494–2.649)	0.755
	I vs. ≥III ^r^	4.554 (1.577–13.148)	**0.005**	1.819 (0.818–4.046)	0.142
	II vs. ≥III ^r^	1.871 (0.777–4.505)	0.162	1.591 (0.712–3.557)	0.258
Neutrophil count (×10^3^) (Per 1 unit increase)		1.229 (0.981–1.539)	0.073	1.168 (0.962–1.416)	0.116
Hematocrit (Per 1 unit increase)		0.895 (0.840–0.954)	**<0.001**	0.991 (0.933–1.052)	0.762
Disease localization (Cutaneous only vs. Extracutaneous involvement present ^r^)		0.419 (0.122–1.441)	0.167	0.515 (0.179–1.482)	0.218
Cutaneous presentation (Localized vs. Disseminated ^r^)		0.533 (0.263–1.082)	0.081	0.552 (0.303–1.007)	0.053
Cutaneous distribution of the lesion					
	Multifocal vs. Localized ^r^	2.701 (1.031–7.077)	**0.043**	3.044 (1.353–6.849)	**0.007**
	Multicentric vs. Localized ^r^	2.743 (1.147–6.557)	**0.023**	2.825 (1.366–5.840)	**0.005**
	Multicentric vs. Multifocal ^r^	1.016 (0.435–2.369)	0.971	0.928 (0.446–1.931)	0.842
Clinical lesion morphology 2 cat (Nodule+Tumor vs. Macule+Plaque ^r^)		0.883 (0.428–1.821)	0.736	1.618 (0.884–2.963)	0.119
Lymphedema (Yes vs. No ^r^)		3.877 (1.422–10.573)	**0.008**	2.385 (0.991–5.743)	0.052
Ulceration (Yes vs. No ^r^)		5.215 (1.522–17.868)	**0.009**	2.749 (0.838–9.022)	0.095
Visceral organ involvement (Yes vs. No ^r^)		5.482 (1.243–24.176)	**0.025**	3.131 (0.741–13.233)	0.121
Overall disease stage					
	III vs. I-II ^r^	1.906 (0.866–4.195)	0.109	2.791 (1.470–5.296)	**0.002**
	IV vs. I-II ^r^	3.879 (1.064–14.141)	**0.040**	4.034 (1.325–12.286)	**0.014**
	IV vs. III ^r^	2.035 (0.551–7.521)	0.287	1.446 (0.479–4.368)	0.514
Primary treatment strategy					
	Systemic therapy only vs. Local therapy only ^r^	0.987 (0.291–3.352)	0.983	1.188 (0.453–3.112)	0.726
	Combined local and systemic therapy vs. Local therapy only ^r^	1.079 (0.407–2.860)	0.878	2.438 (1.199–4.956)	0.014
	Combined local and systemic therapy vs. Systemic therapy only ^r^	1.094 (0.255–4.688)	0.904	2.052 (0.709–5.939)	0.185
Type of local treatment					
	Radiotherapy vs. Surgery ^r^	4.197 (1.415–12.451)	0.01	6.265 (2.397–16.375)	**<0.001**
	RT+Excision vs. Surgery ^r^	2.408 (0.532–10.896)	0.254	2.254 (0.601–8.447)	0.228
	Cryotherapy and laser therapy vs. Surgery ^r^	3.297 (0.596–18.233)	0.172	NE	NE
	RT+Excision vs. Radiotherapy ^r^	0.574 (0.169–1.948)	0.373	0.360 (0.126–1.029)	0.057
	Cryotherapy and laser therapy vs. Radiotherapy ^r^	0.785 (0.181–3.399)	0.747	NE	NE
	Cryotherapy and laser therapy vs. RT+Excision ^r^	1.369 (0.227–8.264)	0.732	NE	NE

Univariate Cox proportional hazards regression was used for OS and PFS. Penalized Cox regression with Firth correction was applied when standard Cox models yielded unstable or non-estimable estimates because of the sparse data or separation. NE indicates estimates that were not estimable because of the sparse data and/or separation. ^r^ Reference category. Bold signifies the statistically significant *p*-values.

**Table 4:** Multivariable Cox proportional hazards models for overall survival and progression-free survival, with adjusted survival probabilities.

			Adjustment Status for Delay from First Symptom to Histological Diagnosis (m) (Maxstat Cut-Off (6.5 m))			
			Unadjusted		Adjusted	
			HR (95% CI)	*p* Value	HR (95% CI)	*p* Value
**Overall survival (OS)**						
	Diagnostic delay (>6.5 vs. ≤6.5)		3.637 (1.644–8.044)	**0.001**	1.711 (0.696–4.207)	**0.242**
	Sex (Male vs. Female ^r^)		3.036 (1.141–8.077)	**0.026**	3.009 (1.117–8.105)	**0.029**
	Age (>70 yr vs. ≤70 yr ^r^)		3.947 (1.675–9.299)	**0.002**	3.057 (1.181–7.914)	**0.021**
	Hematocrit (Per 1 unit increase)		0.892 (0.823–0.967)	**0.005**	0.897 (0.825–0.975)	**0.010**
	Visceral organ involvement (Yes vs. No ^r^)		5.173 (1.085–24.659)	**0.039**	7.335 (1.555–34.61)	**0.012**
	Progression-Free Survival (PFS)					
	Diagnostic delay (>6.5 vs. ≤6.5)		1.542 (0.837–2.843)	**0.165**	1.532 (0.815–2.888)	**0.185**
	Sex (Male vs. Female ^r^)		3.183 (1.341–7.559)	**0.009**	3.137 (1.326–7.421)	**0.009**
	Cutaneous distribution of the lesion					
		(Multifocal vs. Localized ^r^)	2.722 (1.101–6.730)	**0.030**	2.540 (1.039–6.209)	**0.041**
		(Multicentric vs. Localized ^r^)	1.177 (0.390–3.546)	0.773	1.120 (0.372–3.376)	0.840
		(Multifocal vs. Multicentric ^r^)	2.313 (0.863–6.195)	0.095	2.267 (0.832–6.181)	0.110
	Mucosal involvement (Yes vs. No ^r^)		7.894 (1.144–54.447)	**0.036**	9.230 (1.296–65.720)	**0.026**
	Overall disease stage					
		(III vs. I-II ^r^)	3.207 (1.237–8.311)	**0.016**	3.325 (1.276–8.666)	**0.014**
		(IV vs. I-II ^r^)	14.553 (2.462–86.023)	**0.003**	15.945 (2.65–96.06)	**0.003**
		(IV vs. III ^r^)	4.545 (1.013–20.408)	**0.048**	4.796 (1.065–21.60)	**0.041**
			1 year	3 years	5 years	10 years
Adjusted OS % (SE)			93.4 (0.025)	83.9 (0.042)	80.7 (0.047)	44.5 (0.097)
Adjusted PFS % (SE)			89.1 (0.033)	71.5 (0.056)	63.4 (0.063)	20.7 (0.077)

Footnote: Multivariable Cox proportional hazards models were used for OS and PFS. HRs are shown with 95% CIs. ‘Adjusted’ models represent the final parsimonious multivariable Cox models incorporating diagnostic delay categorized by the maxstat-derived cut-off (6.5 months). ^r^ Reference. Abbreviations: OS: overall survival; PFS: progression-free survival; HR: hazard ratio; CI: confidence interval; SE: standard error; yr: years. Variables shown in the adjusted models were selected based on clinical relevance, model interpretability, and sample size and event-per-variable considerations. The adjusted OS model included diagnostic delay group, sex, age group, hematocrit, and visceral organ involvement. The adjusted PFS model included diagnostic delay group, sex, cutaneous distribution, mucosal involvement, and overall disease stage. Adjusted OS and PFS probabilities represent model-based survival estimates calculated at the mean of the covariate values. Bold signifies the statistically significant *p*-values.

## Data Availability

The data supporting the findings of this study are available from the corresponding author upon reasonable request. The data are not publicly available because of privacy and ethical restrictions.

## Supplementary Materials

The following supporting information can be downloaded at: https://www.mdpi.com/article/10.3390/jcm15114243/s1, Table S1. Kaplan–Meier overall survival estimates across diagnostic delay groups and selected clinical subgroups. Footnote: Overall survival was estimated using the Kaplan–Meier method, and group comparisons were performed using the log-rank test. Survival rates are presented as percentages with standard errors (SE).

## Abbreviations

The following abbreviations are used in this manuscript:

KS	Kaposi sarcoma
HHV-8	Human herpesvirus 8
KSHV	Kaposi sarcoma-associated herpesvirus
LANA	Latency-associated nuclear antigen
HIV	Human immunodeficiency virus
AIDS	Acquired immunodeficiency syndrome
STROBE	Strengthening the Reporting of Observational Studies in Epidemiology
ACTG/TIS	AIDS Clinical Trials Group/Tumor–Immune system–Systemic illness

## References

[1] Aydin O., Erciyestepe M., Öztürk A.E., Dinç G., Uysal E., Atci M.M., Ertürk K., Çelik E. (2025). Comparison of clinical and prognostic characteristics of patients with classic and AIDS-related Kaposi sarcoma: A single-center experience. Medicine.

[2] Değerli E., Oruç K., Şentürk Öztaş N., Alkan Şen G., Bedir Ş., Demirci N.S., Demirelli H.F. (2024). Prognostic factors in Kaposi sarcoma, single-centre experience. Australas. J. Dermatol..

[3] Errihani H., Berrada N., Raissouni S., Rais F., Mrabti H., Rais G. (2011). Classic Kaposi’s sarcoma in Morocco: Clinico-epidemiological study at the national institute of oncology. BMC Dermatol..

[4] Park J., Lee J.E. (2024). Localized Radiotherapy for Classic Kaposi’s Sarcoma: An Analysis of Lesion Characteristics and Treatment Response. Cancers.

[5] Valcarcel-Valdivia B., Enriquez-Vera D., Piedra L.E., Holguín A., De la Cruz Ku G. (2023). Treatment outcomes of patients with classic and AIDS-related Kaposi Sarcoma: A single-center real-world experience. Clin. Exp. Med..

[6] McMahon D.E., Chemtai L., Grant M., Singh R., Semeere A., Byakwaga H., Laker-Oketta M., Maurer T., Busakhala N., Martin J. (2022). Understanding Diagnostic Delays for Kaposi Sarcoma in Kenya: A Qualitative Study. J. Acquir. Immune Defic. Syndr..

[7] Brenner B., Weissmann-Brenner A., Rakowsky E., Weltfriend S., Fenig E., Friedman-Birnbaum R., Sulkes A., Linn S. (2002). Classical Kaposi sarcoma: Prognostic factor analysis of 248 patients. Cancer.

[8] De Boer C., Niyonzima N., Orem J., Bartlett J., Zafar S.Y. (2014). Prognosis and delay of diagnosis among Kaposi’s sarcoma patients in Uganda: A cross-sectional study. Infect. Agent. Cancer.

[9] Von Elm E., Altman D.G., Egger M., Pocock S.J., Gøtzsche P.C., Vandenbroucke J.P. (2007). Strengthening the reporting of observational studies in epidemiology (STROBE) statement: Guidelines for reporting observational studies. BMJ.

[10] Lausen B., Schumacher M. (1992). Maximally Selected Rank Statistics. Biometrics.

[11] Hothorn T., Lausen B. (2003). On the exact distribution of maximally selected rank statistics. Comput. Stat. Data Anal..

[12] Firth D. (1993). Bias Reduction of Maximum Likelihood Estimates. Biometrics.

[13] Heinze G., Schemper M. (2001). A solution to the problem of monotone likelihood in Cox regression. Biometrics.

