# Supplementary material for: Longer Diagnostic Delay and Post-Diagnosis Overall Survival in a Classic-Kaposi-Sarcoma-Predominant Retrospective Cohort
Source: J Clin Med. 2026 May 30;15(11):4243. doi: 10.3390/jcm15114243 (PMC13258345; doi:10.3390/jcm15114243)
Supplement: Supplementary file 1 [file jcm-15-04243-s001.zip › jcm-4253736-supplementary.pdf]

Supplementary Table S1: Kaplan–Meier overall survival estimates across diagnostic delay groups and selected clinical subgroups.

	Deaths, n (%)	Alive at last follow-up, n (%)	Estimated overall survival at each time point				P value
			1 year (n=75)	3 years (n=53)	5 years (n=36)	10 years (n=10)	
	n (%)	n (%)	OS (%) (SE)	OS (%) (SE)	OS (%) (SE)	OS (%) (SE)	
**Diagnostic delay category**							**<0.001**
Early (n=46)	10 (21.7)	36 (78.3)	97.8 (0.022)	90.0 (0.048)	90.0 (0.048)	68.3 (0.104)	
Late (n=41)	21 (51.2)	20 (48.8)	82.6 (0.060)	69.2 (0.075)	62.2 (0.082)	19.4 (0.112)	
**Sex**							0.367
Female	9 (40.9)	13 (59.1)	90.9 (0.061)	90.9 (0.061)	85.2 (0.080)	50.5 (0.147)	
Male	22 (33.8)	43 (66.2)	90.6 (0.037)	75.9 (0.057)	73.4 (0.060)	36.9 (0.115)	
**Visceral involvement**							**0.011**
No	29 (34.9)	54 (65.1)	92.7 (0.029)	81.6 (0.045)	78.0 (0.050)	46.2 (0.089)	
Yes	2 (50.0)	2 (50.0)	75.0 (0.217)	37.5 (0.286)	37.5 (0.286)	37.5 (0.286)	
**Age**							**<0.001**
≤70 years	10 (21.3)	37 (78.7)	95.4 (0.032)	95.4 (0.032)	95.4 (0.032)	57.6 (0.125)	
>70 years	21 (52.5)	19 (47.5)	82.4 (0.060)	61.7 (0.082)	57.8 (0.085)	18.0 (0.108)	
**Total**	31 (35.6)	56 (64.4)	90.7 (0.031)	80.0 (0.045)	74.4 (0.052)	45.3 (0.087)	

Footnote: Overall survival was estimated using the Kaplan–Meier method, and group comparisons were performed using the log-rank test. Survival rates are presented as percentages with standard errors (SE).
